# Group 2 Innate Lymphoid Cells in Pulmonary Immunity and Tissue Homeostasis

**DOI:** 10.3389/fimmu.2018.00840

**Published:** 2018-04-30

**Authors:** Barbara C. Mindt, Jörg H. Fritz, Claudia U. Duerr

**Affiliations:** ^1^Department of Microbiology and Immunology, McGill University, Montreal, QC, Canada; ^2^McGill University Research Centre on Complex Traits (MRCCT), McGill University, Montreal, QC, Canada; ^3^FOCiS Centre of Excellence in Translational Immunology (CETI), McGill University, Montreal, QC, Canada; ^4^Department of Physiology, McGill University, Montreal, QC, Canada; ^5^Institute of Microbiology and Infection Immunology, Charité – University Medical Centre Berlin, Berlin, Germany

**Keywords:** group 2 innate lymphoid cells, respiratory tract, innate immune responses, lung physiology, pulmonary microenvironment

## Abstract

Group 2 innate lymphoid cells (ILC2) represent an evolutionary rather old but only recently identified member of the family of innate lymphoid cells and have received much attention since their detailed description in 2010. They can orchestrate innate as well as adaptive immune responses as they interact with and influence several immune and non-immune cell populations. Moreover, ILC2 are able to rapidly secrete large amounts of type 2 cytokines that can contribute to protective but also detrimental host immune responses depending on timing, location, and physiological context. Interestingly, ILC2, despite their scarcity, are the dominant innate lymphoid cell population in the lung, indicating a key role as first responders and amplifiers upon immune challenge at this site. In addition, the recently described tissue residency of ILC2 further underlines the importance of their respective microenvironment. In this review, we provide an overview of lung physiology including a description of the most prominent pulmonary resident cells together with a review of known and potential ILC2 interactions within this unique environment. We will further outline recent observations regarding pulmonary ILC2 during immune challenge including respiratory infections and discuss different models and approaches to study ILC2 biology in the lung.

## Introduction

Group 2 innate lymphoid cells (ILC2) have been identified less than a decade ago and constitute a new member of the family of innate lymphoid cells (1–3). From an evolutionary aspect, ILC2 are thought to be a rather old cell type with ancestor populations proposed in lamprey and bony fish (4). The indication of an innate lymphoid cell population linked to a type 2 immune response was first made in the 2000s: in mice, an IL-25-inducible non-T non-B cell population was reported to release large amounts of IL-5 and IL-13 (5), and in humans, a CD34^+^ population expressing both, IL-33R and TSLPR, was identified and shown to secrete type 2 signature cytokines upon stimulation with IL-33 or in combination with TSLP (6). In 2010, ILC2 were eventually described in detail and were discovered to reside in distinct tissues in mice and termed nuocytes, natural helper cells, or innate helper cells (3, 7, 8). Despite the identification of ILC2 in various anatomical sites such as adipose tissue, liver, mesenteric lymph nodes (LNs), and the small intestinal lamina propria, common characteristics of ILC2 became apparent from these early studies. Those include their cytokine receptor expression profile, signature cytokines that are released, as well as their characteristic transcription factors. Thus, nomenclature of ILC2 was unified rather soon after their discovery (9). Until now, ILC2 have been described in mice and humans at varying body sites but with many overlapping characteristics: ILC2 share the ability to produce large amounts of type 2 cytokines within a short period of time and have common basic phenotypic characteristics regarding maintenance, regulation of surface molecule (e.g., CD127 and CD25) and transcription factor (e.g. GATA3) expression (9). However, it was also observed quickly that some variations in their typical phenotypic profile exist depending on the anatomical site and/or maturation level (e.g., expression of c-kit) (10). The location of ILC2 is not limited to mucosal sites, but due to their innate character and fast and immediate action, ILC2 are thought to be especially important at barrier surfaces. For example, in pulmonary immunity, ILC2 play a non-redundant role and are able to trigger airway hyper-reactivity even in the absence of adaptive immunity (7). However, it is clear that ILC2 are not isolated in their action and that their function and physiological role is regulated by and regulates other pulmonary hematopoietic and non-hematopoietic cells. In contrast to T helper 2 (Th2) cells, their adaptive counterparts, current evidence supports the notion that ILC2 are tissue resident and thereby act as key players within their corresponding tissue microenvironment (11, 12). Although all ILC types can be found at the mucosal surface of the small intestine, ILC2 are the predominant ILC population in the lungs at steady state. The reason for this skewed distribution of ILC populations in the lungs is not yet fully understood. However, the lungs provide without a doubt a unique microenvironment for ILC2.

In this review, we therefore focus on the pulmonary microenvironment, its physiology regulated by non-hematopoietic resident cells, and how ILC2 functionality is embedded within. We will further discuss different pulmonary infection and cytokine challenge models in which ILC2 effector functions have been shown to play important roles. We will only briefly address plasticity of ILC2 and their regulation since both have been recently discussed in an excellent review (13).

## Lung Physiology and the Pulmonary Microenvironment of ILC2

**Figure 1:**
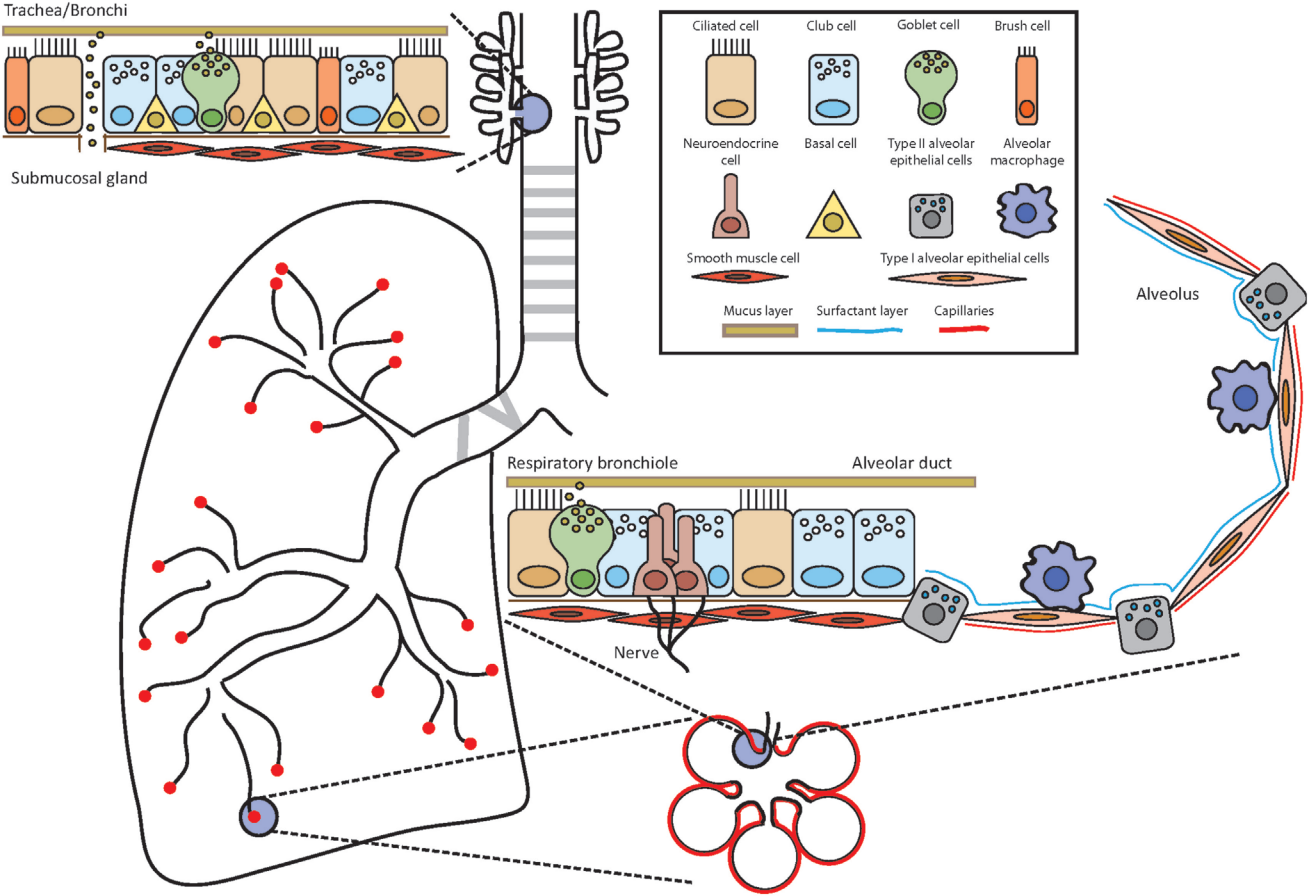
Anatomy of the airways. Throughout the airways, the cell composition of the epithelium changes to ensure optimal and efficient gas exchange as well as maintenance of lung integrity and defense against potential pathogens and allergens. Depending on the location, the epithelium is composed of several different cell types such as ciliated cells, club cells, brush cells, goblet cells, airway smooth muscle cells, neuroendocrine cells, type I and type II alveolar epithelial cells, and alveolar macrophages. Basal cells have the potential to differentiate into several lineages and serve as stem cells.

Group 2 innate lymphoid cells have been isolated from and were described in various tissues of the respiratory system. These reports include ILC2 from both mouse and human lungs (3, 7, 14–16) and adjacent sites such as human nasal and tonsil tissues (17). We will therefore give a short overview of our respiratory system and the main populations of tissue-resident cell populations focusing mainly on non-hematopoietic cells (Figure 1).

Our respiratory system can be divided into the upper and lower airways. The upper airways span from the nose (nasal cavity/nasopharynx) to the pharynx (oropharynx and laryngopharynx) and further down to the trachea and bronchi. The bronchi branch into the smaller terminal and subsequently into the respiratory bronchioles from which the alveolar ducts are generated and end in the alveolar sacs consisting of several alveoli. The alveolar ducts and the alveoli compose the lower airways. The main responsibility of the respiratory system including these different parts is to ensure efficient gas exchange and thereby provide oxygen to the organism in exchange for carbon dioxide. To accomplish this, air is inhaled at a rate of several liters each minute in the resting state and delivered to this network of branching tubes. The blind-ended alveoli are the place of efficient exchange of carbon dioxide to oxygen. All other parts of the airways can be envisioned in an oversimplified way as a conduit, which serves for the airflow to reach the alveoli. However, the delivery of inhaled air through this conduit, which may be loaded with potential antigens, allergens, and pathogens, is controlled by mechanical filters, physiological barriers, active expel, and immune defense mechanisms (18). These tasks are accomplished by the interaction and interplay of approximately 40 distinct cell populations in the lungs (19). The well-organized and delicate structure of our respiratory immune system and its immediate surrounding is therefore a unique microenvironment for ILC2.

### The Upper or Conducting Airways

The upper airways span from the nose and trachea to the bronchi (primary, secondary, and tertiary) and further down to the bronchioles (terminal and respiratory). The lungs start at the first branching point of the bronchi from the trachea. The character of the tissue of the bronchi still resembles the trachea with a pseudostratified, ciliated columnar epithelium including goblet cells, mucus glands, and cartilage. The cells of the epithelial layer are tightly interlinked *via* adhesion junctions composed of tight junctions, adherens junctions, and desmosomes, thereby establishing a firm physical barrier (20, 21). Throughout the branching of the tubular network, the epithelium changes its composition from the characteristic ciliated columnar epithelium in the trachea and larger bronchi to a mix of non-ciliated and ciliated cells in the bronchioles and terminal bronchioles to the respiratory bronchioles with rare occurrence of ciliated cells (18). Several different epithelial and non-epithelial lung resident cells will be discussed below.

#### Goblet Cells

Goblet cells are defined by their goblet or cup-like shape that is acquired by their inner cellular structure of secretory granulae filled with mucins (22). Goblet cells are present in humans in the trachea and bronchi as well as in the submucosal glands at steady state and absent in the smaller branching of the airways. However, they can be induced upon infection or challenge. In the non-challenged laboratory mouse, goblet cells are mainly restricted to submucosal glands but can be induced upon challenge in trachea, larger bronchi, and even in the bronchioles. The distribution of goblet cells is an important difference between mouse and human physiology (23). IL-13 is key for goblet cell hyperplasia and function such as mucus secretion (24, 25) and is also one of the signature cytokines that is produced and released by ILC2 (26).

#### Club Cells

Club cells or bronchiolar exocrine cells have characteristic short microvilli, a dome shape, and are present in bronchioles (terminal to respiratory). Club cells secrete surfactant proteins (surfactant protein A, B, and D) and express Clara cell 10 kDa protein (CC10, *Scgb1a1*), which can bind to surfactant lipids. Club cells are able to self-renew but also to differentiate into ciliated cells to regenerate the epithelium (27) as well as into goblet cells (28).

#### Pulmonary Neuroendocrine Cells (PNECs)

Pulmonary neuroendocrine cells are a rare cell population, which represent only approximately 1% of the airway epithelium. PNECs are present in clusters, termed neuroepithelial bodies, and are located at airway branching points. They are an innervated epithelial cell population (29), sense changes in oxygen levels, and release neuropeptides (calcitonin gene-related peptide), neurotransmitters (serotonin), and bombesin-related peptide (Neuromedin B) (30). In addition, PNECs play a role in the modulation of smooth muscle tonus. Since PNECs transmit environmental signals *via* the rich network of neural fibers (postganglionic parasympathetic neurons and the vagus nerve) to the central nervous system, they serve as a link between the nervous and endocrine system. A role of PNECs in immune responses and tissue remodeling has been recently reported (31) and deregulated PNECs are associated with different respiratory diseases such as chronic obstructive pulmonary disease (COPD) (32) or asthma (33).

#### Brush (Microvillous) Cells

The role of brush cells (Tuft cells, caveolated, multivesicular, and fibrilovesicular cells) in normal airways and alveoli is poorly understood yet, albeit their existence has been known for some time (19). Brush cells are pear or flask-like shaped cells (wide base and narrow microvillous apex) with a tuft of blant and broad, squat microvilli. They have first been described in the airway epithelium and later in alveoli (alveolar lining) as the third pneumocyte in addition to type I and type II airway epithelial cells (34). In humans, brush cells exist from the nose to the alveoli, but are only present in alveoli in disease states (35). In the mouse, brush cells are abundant in the trachea (36). Interestingly, brush cells express (bitter) taste receptors and are able to regulate breathing by signal transmission to neurons of the vagus nerve (36, 37). Brush cells are not only present in the mucosa of the respiratory tract but also in the small intestine. Here, brush cells are termed tuft cells and have recently been shown to constitute an important source of the ILC2-stimulating cytokine IL-25 (38–40). Furthermore, IL-13 secreted by ILC2 is able to induce tuft cell hyperplasia in the small intestine, indicating a positive feed forward loop (38–40). However, although IL-25 can be detected in pulmonary tuft cells as well, it is currently unknown whether a similar regulatory interaction between tuft cells and ILC2 is also of functional relevance in the lungs. Upon intranasal administration, IL-25 is significantly less potent in eliciting a pulmonary type 2 immune response compared with IL-33 and also does not increase pulmonary ILC2 (41, 42). The mode of IL-25 administration (intranasal vs systemic) is also key in eliciting different ILC2 populations in the lung (43, 44) and as such it is not known yet if and to what extent brush cells are involved in induction of different pulmonary ILC2 subgroups.

#### Nerve Cells (Neurons)

Neurons have a very characteristic shape due to their function: their cell body is surrounded by dendrites to carry (electrical) impulses to the cell and a long axon is used to transfer impulses to other cells. Impulses are transmitted *via* the synapse, a gap between the axon of one neuron and the dendrites of another neuron. The upper and lower airways are innervated with neurons of nerves such as the vagus nerve (pneumogastric nerve, cranial nerve X) emerging from the base of the brain to the chest and lungs, and then passing the heart to the colon. The vagus nerve belongs to the parasympathetic division of the (peripheral) autonomic nervous system, which controls involuntary processes such as the regulation of the heart and respiratory rate (*via* smooth muscle cells) as well as the orchestration of glands and mucous secretion (45). The axons of the vagus nerve’s neurons branch off into all parts of the airways. Vagus nerve stimulation leads to constriction of the airways and is essential for actual breathing (46). Spinal nerves transmit impulses between the spinal cord and the body. Several different spinal nerves are present in the body amongst them are the twelve pairs of thoracic nerves, which emerge from the thoracic vertebrae and are further divided into proximal and distal branches. Clusters of neuron cell bodies (soma) make up a ganglion and can be identified as swellings along nerves. In addition, impulses for defense mechanisms are initiated such as coughing, which is often deregulated and/or dysfunctional in airway diseases (47, 48). The neuro-immune axis is increasingly becoming the central focus of current pulmonary research and a link between ILC2 function and neurons has been described just recently (see below).

#### Airway Smooth Muscle Cells (ASM)

Airway smooth muscle cells cover the complex branching network of the bronchial tree in a circumferential fashion and perpendicular to the axis of the tube (49). At the start of the trachea, ASM lie in an almost complete sheet of smooth muscle in the posterior wall of the trachea at the opening of the C-shaped cartilage. This almost complete ring of ASM breaks down in the main bronchi between the ends of the U-shaped cartilage rings. In the subsequent bronchi, ASM form discontinuous bands between outside extremities of each cartilage ring. These muscular walls with cartilage rings extend from the larynx over the primary bronchus to the smaller bronchi in humans. From the bronchioles onward, the smooth muscle fibers lie between adventitial tissue and epithelium and the cartilage decreases with the presence of these incomplete muscular walls. In mice, no cartilage plates are present in the lower airways, and ASM muscle bundles describe helical patterns from the medium bronchi to the alveolar duct. Thereby, ASM build an important support structure for the lungs and prevent its collapse. Moreover, ASM are key for the contraction of the airways. But ASM may also play an important immunological role in the lungs with the expression of immune mediators and cytokines or their expression of (bitter) taste receptors (50). The implication of ASM in asthma has been reported almost a century ago since ASM mass is increased in asthma patients (51). However, the exact contribution and function of ASM in respiratory diseases, their cytokine secretion, induction of proliferation, as well as their potential interaction or competition with other pulmonary cell populations still needs to be elucidated in detail.

#### Stem Cells

The lungs show a low regenerative rate at steady state in contrast to other mucosal tissues such as the small intestine. However, upon infection or challenge, the lungs are able to initiate a high-regenerative turnover program (52). Basal cells have been described as the putative stem cells, are shared between bronchioles and alveoli, and have been suggested to generate ciliated, mucous, and neuroendocrine cells (23). In 2005, these basal cells were termed bronchioalveolar stem cells and their importance for lung regeneration but also for cancer was described (53). Recently, two reports identified two slightly different stem cell populations in mice upon influenza virus infection (54, 55). These stem cells have epithelial characteristics with the formation of Krt-5^+^ cell clusters (pods) as an important and characteristic feature. Interestingly, these cells are able to migrate from a distal position to the injured tissue area, where they serve as progenitors for different cellular lineages such as type I and type II pneumocytes (54, 55). However, repair mechanisms in human lungs are far less understood than in mice.

#### Submucosal Glands

Mucus glands are present in the lamina propria of the trachea and bronchi but absent in the distal tubular network (18). Their lamina propria is a loose and collagen rich structure, which supports the pseudostratified columnar epithelium of the trachea and bronchi.

### Lower or Respiratory Airways

The alveoli of the lower airways are the place where oxygen is drawn and carbon dioxide is discarded. Whereas no cartilage is present in the alveolar duct and alveoli, smooth muscle cells are quite rare in the alveolar duct and absent in the alveoli. The delicate organization of the alveoli provides the perfect physiological anatomy to ensure this exchange. The alveoli are lined by type I and type II alveolar epithelial cells (pneumocytes). Type I cells are flat and spread out and thereby cover approximately 90% of the alveolus’ surface area even if they are not the most frequent cell population in numbers. Thereby, they provide a thin cellular layer, which serves as an optimal respiratory surface. Importantly, type I cells are not able to divide but can be restored by type II cells differentiating into type I cells (56). Type II cells are of cuboid shape and have characteristic microvilli on their apical surface and lipid rich lamellar bodies containing surfactant proteins. Surfactant proteins cover the alveolus surface in a thin layer and are important to prevent a collapse of the alveoli during breathing but also exert antimicrobial activity (57). Type II cells also produce cytokines such as TNFα (58). Alveolar macrophages are situated in close proximity to type I or type II cells lining the alveolus as well as in the alveolar space. Moreover, type I cells are in close contact to the endothelial cells of the alveolar capillary which are key for gas exchange. The alveolar capillaries are circumventing alveoli with a network so that every capillary is lining two alveoli, one from each side, thereby an optimal gas exchange is guaranteed. The alveolar capillaries are present within the septae, which separate the alveoli from each other. Interestingly, each alveolus is not isolated but connected to adjacent alveoli by discrete holes, termed the pores of Kohn or interalveolar connections (59). These alveolar pores have been proposed to play a role in ensuring that the lungs do not collapse and equalize pressure. They are important in collateral ventilation and ensure minimal ventilation even if the lung partially deflates. However, other observations suggest that these pores are not empty (60), which could indicate that these interalveolar connections allow spreading of small particles and other infectious material such as bacteria or viruses. Moreover, it has been suggested that immune cells such as alveolar macrophages are able to migrate through these pores (61). It is tempting to speculate that upon challenge pulmonary ILC2 might use these pores as a passageway to get from one alveolus to another and thereby survey the tissue.

The organization of human and mouse lungs is very similar in general; however, several important differences exist, and some of them have been already mentioned in this review. For the sake of completeness, we would like to point out that unlike in humans, the mouse lung consists of five lobes: the left lung, and the right lung with the superior, middle, inferior, and post-caval lobes. By contrast, the human lung is composed of the two lobes (upper and lower lobe) of the left lung, and three lobes of the right lung (upper, middle, and lower). In addition, the branching pattern of the airways is less complex in mice compared to humans (62, 63), with the predominant cell types of the conducting airways being ciliated cells in humans, whereas more secretory cells are present in mice (64, 65).

## Definition and Characteristics of Pulmonary ILC2

The definition of pulmonary ILC2 was shaped by their first identification in cytokine reporter mice. Here, pulmonary ILC2 were defined by their respective reporter signal in IL-4 (4get) and IL-13 (YetCre-13) reporter animals (3). Later on, ILC2 were described in the lungs of non-reporter and wild-type mice. Although the gating strategies vary, ILC2 are in general defined as lineage negative cells, which are positive for Thy1, Sca-1, GATA-3, T1/ST2, inducible T cell costimulator (ICOS), CD44, CD25, CD127, KLRG1, and c-kit low to positive (7, 14, 66–68). These characteristics of pulmonary ILC2 also are consistent with ILC2 at other mucosal sites such as the gut (2, 67, 69). To the best of our knowledge, there is so far no sole known (surface) marker or characteristic that defines ILC2 at one site but is not present or inducible at any other site. This is supported by the current concept that ILC2 represent one group of innate lymphoid cells, but that this group is not always homogenous. Single-cell sequencing analysis of ILC2 cells at steady state in the small intestine showed that ILC2 are indeed heterogeneous and several subgroups with different gene expression patterns were identified (70). Moreover, an approach using signature type 2 cytokine reporter mice revealed that pulmonary ILC2 exhibit different mRNA expression patterns of these cytokines when compared with small intestinal ILC2. *Il5* is expressed at steady state in both pulmonary and intestinal ILC2, whereas *Il13* is continuously expressed in small intestinal ILC2, but can be induced in pulmonary ILC2 upon challenge (71). In the lungs, inflammatory (i) and natural ILC2 (nILC) have been reported upon systemic administration of IL-25 or IL-33. Although these ILC2 subgroups are well defined by their slightly different surface markers, namely high killer cell lectin-like receptor subfamily G member 1 (KLRG1) expression for iILC2 and ST2 expression for nILC2, a transition has been noted between iILC2 and nILC2, highlighting a strong relation of these two populations (43). These reports underline that ILC2 belong to one group within the ILC family but that ILC2 biology and characteristics are highly influenced and shaped by their respective microenvironment, its immunological profile at steady state as well as upon immune challenge.

Our knowledge of human pulmonary ILC2 is limited at the moment due to the fact that the accessibility of human lung samples is highly restricted. The information about human ILC2 originates from deceased patient material (organ donors) or from surgeries of the lungs such as routine bronchoscopy upon lung transplantation. In those tissues, ILC2 can be detected and defined as lineage negative, CD127^+^ CD25^+^ and ST2^+^ (14) and thereby share important characteristics with mouse ILC2. In lungs of idiopathic pulmonary fibrosis and COPD patients, ILC2 were identified as lineage negative, CD127^+^ ST2^+^ ILC2, or lineage negative, CD127^+^ CRTH2^+^ ILC2. ST2^+^ ILC2 show high expression of arginase 1, which was identified in this elegant study as an important characteristic for ILC2 functionality (15). Human ILC2 obtained from patients undergoing lung tumor surgery can be identified as CD45^+^ and CD127^+^ cells upon excluding lineage positive cells. Interestingly, compared with ILC2 from nasal polyps of chronic rhinosinusitis patients, these pulmonary ILC2 exhibit differences in their pattern of CRTH2 (chemoattractant receptor-homologous molecule expressed on Th2 cells) and c-kit expression (16), which might be due to their distinct microenvironment. Furthermore, CRTH2 was recently identified to play a role in ILC2 accumulation in the lungs of human and mice, and differences in CRTH2 expression between human peripheral blood (high) and pulmonary (low) ILC2 (lineage^-^CD127^+^CD45^+^ST2^+^) may as such determine tissue localization and function (72). These data underline that human and mouse ILC2 share biologically important similarities.

## Tissue Residency and Local Distribution of Pulmonary ILC2

In the lungs, the presence of hematopoietic cells at steady state is relatively low compared with other mucosal sites such as the small intestine. However, B and T cells [including T regulatory (Treg) cells], alveolar and interstitial macrophages, different types of dendritic cells, and ILC can be detected in addition to resident, non-hematopoietic cells (described above). Interestingly, ILC2 are the predominant ILC population in the lungs at steady state (14). Although the localization and migration capacity of ILC2 determine their (potential) interaction partners, we are only just beginning to understand these dynamics.

Based on observations made by two recent studies using a parabiosis approach, ILC2 are regarded as tissue-resident cells. No significant numbers of ILC2 of the parabiont host were found in the lungs within 4 months of parabiosis (11). Strikingly, even within one week post *Nippostrongylus brasiliensis* infection, only host ILC2 were detected in the lungs, underlining the tissue residency of ILC2. However, two weeks post-infection a significant increase of donor-derived ILC2 were detected in lung, small intestine, and mLN, while no changes were observed for ILC3 in the small intestine. This might reflect the limited local proliferation of the small and insignificant number of donor ILC2 during the infection. In another elegant study, Koyasu and colleagues analyzed parabiotic pairs after more than one month of shared circulation for pulmonary T cells, ILC2, and eosinophils at steady state, or up to one week post intratracheal IL-33 challenge. Whereas T cells and eosinophils are present from donor and host in this parabiotic pair, ILC2 of the parabiotic donor could not be detected (12), underlining that ILC2 remain local and do not migrate into the parabiotic host. Both reports elegantly show and strengthen the current notion that ILC2 are tissue-resident cells that primarily retain their tissue residency at steady state as well as during immune challenge. Interestingly, ILC2 have been shown to accumulate in the mouse lungs during ontogeny with ILC2 numbers peaking at about two weeks after birth (73, 74). The ILC2 of these immature mice exhibit increased levels of intracellular cytokines when compared with ILC2 in adult animals and are to some extent dependent on IL-33. Upon challenge with house dust mite (HDM), more IL-33 protein can be detected in lungs of immature mice compared to adult mice accelerating innate as well as adaptive type 2 immune responses (73, 74). However, further research will be needed to better understand how ILC2 are directed to find their respective tissue niches during ontogeny. Moreover, since ILC2 are observed in lungs at steady state and upon challenge, the question remains where within the tissue these cells are exactly located, how they take part in tissue surveillance and which cellular interactions with other resident and infiltrating cells are established, supported, and needed for maintenance and function in acute and chronic lung diseases.

In addition, it will be of great interest to understand how and under which circumstances ILC2 egress from the bone marrow as well as how they migrate between distinct peripheral tissues. A recent study by Stier and colleagues sheds light on the migratory ability of ILC2. They observed that IL-33 is key in regulating ILC2 egress from the bone marrow (75) since ILC2P in IL-33-deficient mice show similar fitness to wild-type mice but are retained in the bone marrow due to higher expression of CXCR4. In addition, by using an elegant combination of parabiosis and tissue injury, they show that ILC2 are capable of migration after sublethal irradiation and thereby repopulating the empty ILC2 niche. This report demonstrates that under specific circumstances ILC2 are indeed able to traffic, even if mainly regarded as a tissue-resident population. In this report, ILC2 are identified by their expression of the IL-25 receptor chain (IL-25R or IL-17RB), which identifies ILC2 progenitors in the bone marrow (76) but limits detection to iILC2 (43) as well as memory ILC2 (77) in peripheral tissue. If this holds true for ILC2 in general including nILC2, which are in contrast identified based on lower levels of KLRG1 and alternatively by GATA3 expression, still needs to be elucidated. Another elegant report using parabiotic mice identifies that iILC2 are able to migrate from the small intestine to the lungs upon systemic (i.p.) IL-25 administration or helminth infection (78). This study further shows that especially intestinal iILC2 have the potential to migrate and that this migration is regulated in a S1P-dependent manner. Interestingly, upon systemic IL-25 challenge, nILC2 reside in the alveolar space in contrast to iILC2, which are confined to the vascular space.

In addition, S1PR1 (*S1pr1*) and L-selectin (CD62L, *Sell*) expression was detected in naïve lung ILC2 and decreased upon stimulation with IL-33 (77). The adaptive counterpart of ILC2, T cells are guided by S1PR1, which binds to S1P1, to exit secondary lymphoid organs and the thymus (79). Moreover, ILC2 have been detected in the mesenteric LN upon systemic challenge (2) but also in the mediastinal LN of the lungs upon intranasal administration of IL-33 or papain (77). This might be due to proliferation of local tissue-resident ILC2 or active migration. However, the ability of homing of ILC2 from distal sites, such as the lung or small intestinal tissue, to the respective draining LNs is only beginning to be understood. In an elegant study using *Kaede* transgenic mice in which distinct cell movements can be tracked *via* a photoconvertible fluorescent protein, Mackley and colleagues show that all ILC populations constitutively traffic from the small intestine to the mLN (80). Interestingly, this migration is only dependent on the CCR7 receptor for LTi-like ILC3, but not for ILC1 or ILC2 (80). Moreover, opposite trafficking from the mLN to the small intestine is dependent on CCR7 for ILC1 and ILC3 and their further migration to the small intestine is regulated by a retinoic acid regulated homing receptor switch in contrast to ILC2 (81), further indicating that different homing programs exist for the different ILC populations. Importantly, immunofluorescence analysis revealed that ILC2 and ILC3 are located in close proximity to each other, within the interfollicular space, in the mLN (80). Whereas both of these ILC populations as well as ILC1 are present in all investigated LNs, this close proximity of ILC3 and ILC2 was only observed in the mLN (80). Interestingly, ILC3 are most prominent in wild-type mice in the mLN, but the ratio is shifted to higher levels of ILC2 in Rag-deficient mice (80). How this imbalance is caused has not been fully understood but a different activation and metabolic state in ILC3 in Rag1-deficient mice compared with wild-type mice has been recently reported (82) and this might in addition interfere with the trafficking ability of ILC3. However, whether communication between ILC2 and ILC3 in the special microenvironment of the mLN and in draining LNs exists and if this is a general phenomenon still needs to be determined.

Thus, ILC2 might support immune reactions not by circulating through the blood to reach peripheral tissues, but by traveling rather short distances to a respective LN. It is tempting to speculate that ILC2 might have a special function within the elicited immune response at a defined location similar to what has been described for natural killer (NK) cells (83). However, the importance of the microenvironment for ILC2 in the mediastinal LN and the regulatory mechanisms of homing to and from it as well as to the lungs still need to be elucidated.

In contrast to mice, ILC2 have been identified in the blood of humans since their detailed description (16). Lombardi et al. investigated patients with allergic rhinoconjunctivitis with or without asthma and healthy, non-allergic individuals (84). Surprisingly, the number of ILC2 as well as cytokine production profile is similar between healthy donors and allergic subjects. However, ILC2 are slightly increased in asthmatic patients compared with non-asthmatic within the group of allergic subjects. However, this trend is also observed with peripheral ILC3. mRNA sequencing comparing allergic and non-allergic subjects revealed an increase of genes involved in the activator protein 1 molecule and suggests a higher activity of its related pathway. Importantly, surface expression of the CCR10 receptor on ILC2 was significantly elevated in allergic compared with non-allergic individuals and expression of CCR10 can be correlated to increased severity of asthma. However, no change was observed in CCR4 expression in peripheral blood of allergic and non-allergic individuals (84).

Since ILC2 are the dominant population of ILC in the lungs, it is important to determine their location(s) to understand their biology, cellular interaction partners, as well as the influence of the pulmonary microenvironment on their function. The first report on the localization of pulmonary ILC2 identified them in collagen rich structures in close proximity to the airways at steady state by using an IL-5 reporter mouse (71). This location was further defined to be close to the epithelium and small conducting airways (8). Upon IL-33 challenge, pulmonary ILC2 are often situated in clusters that are located in the vicinity to the airway epithelium as well as in the alveolar space. The formation of these ILC2 clusters is not limited to cytokine administration and has been also detected in alveolar spaces of helminth-infected mice (*N. brasiliensis*, two weeks post-infection) (8). Interestingly, clusters of ILC2 are situated close to infected foci/cells of bronchioles and alveoli upon influenza infection (ST2-GFP-reporter mice) (85). Upon pneumectomy, ILC2 are induced and have been identified in the conducting airways and in the alveolar space using IL-5 reporter mice (86). Recent reports further identified that ILC2 are located in the vicinity of (enteric) neurons (41, 87).

In summary, ILC2 are located at peripheral and central sites of the lungs at steady state and are present in clusters within affected cellular foci upon challenge. The appearance of these ILC2 clusters is interesting and raises the question of whether ILC2 actively proliferate at these locations due to stimuli provided by their infected microenvironment or/and if they migrate from more peripheral locations toward these infected foci. Moreover, it will be of interest to decipher whether pulmonary ILC2 are able to keep the lung tissue under surveillance similar to skin ILC2 (88), and thereby actively contribute to the homeostasis of the lungs. In addition, passing and migrating through the pores of Kohn would be a huge advantage to reach alveoli as speculated above and as was proposed for macrophages (61). However, how and if these migration activities of ILC2 are directed still remains elusive.

## Plasticity of Pulmonary ILC2

Group 2 innate lymphoid cells have been originally identified in different organs under different conditions and with slightly different characteristics. The key characteristics of ILC2, which have led to their standardized nomenclature were mainly the expression of the transcription factor GATA3, the expression of surface receptors such as ST2, and the production of type 2 signature cytokines. However, it has been observed that ILC2 are able to adapt due to different challenges and microenvironments. This plasticity of ILC2 changes the typical ILC2 characteristics such as transcription factor expression, cytokine expression, and surface receptor expression or a combination thereof. Upon IL-25 challenge, iILC2, which have migrated from the small intestine, are observed in the lungs (43, 78). iILC2 express both GATA3 and RORγt and have the potential to express IL-17 besides IL-13 in *ex vivo* culture experiments, thereby iILC2 can as well support antifungal immune responses. In addition, iILC2 exhibit plasticity by transitioning to nILC2 characterized by ST2 instead of IL-17RB surface expression under certain conditions (43).

Importantly, ILC2 downregulate the key transcription factor GATA3 upon respiratory challenges of viral or bacterial origin as well as environmental pollution such as cigarette smoke. These converted ILC2 are able to secrete IFN-γ in response to IL-12 and IL-18 (85). Of note, human ILC2 are able to adapt to this ILC1-like phenotype when cultured with IL-12 (89). Moreover, the induction of this conversion in human ILC2 is initiated by IL-1β (89, 90). Interestingly, IL-1β in combination with IL-2 is a strong activator of human peripheral blood ILC2 and signals through NF-κB (89, 90). Thus, ILC2 plasticity is of high importance regarding the development of therapeutics to counter pulmonary type 2 immunopathologies.

## Cell Surface Receptor–Ligand Interactions of ILC2 with other Cells of the Pulmonary Environment

**Figure 2:**
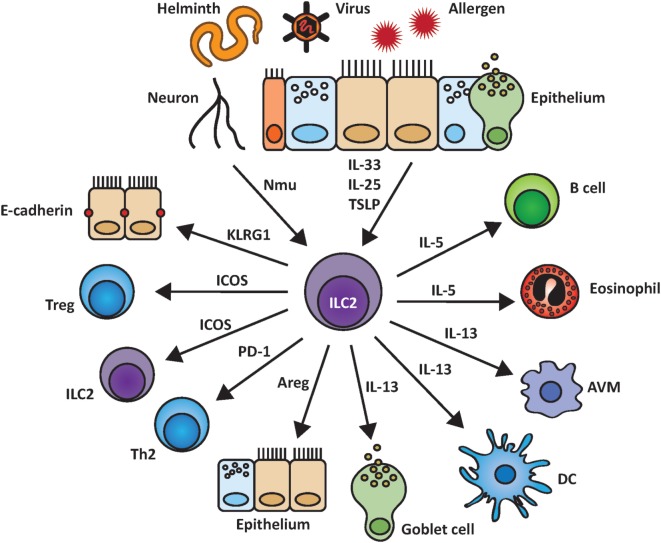
Group 2 innate lymphoid cells (ILC2) and their interactions within the pulmonary environment. ILC2 are induced by epithelial-derived cytokines as well as by other biological mediators such as the neuropeptide neuromedin U (NMU). Upon activation, ILC2 are able to stimulate and communicate with other cell populations by indirect cytokine based or cell–cell mediated interactions including the release of IL-5, IL-13, and amphiregulin (Areg) and binding of KLRG1 to E-cadherin.

Group 2 innate lymphoid cells express various surface molecules that can interact with their respective ligands on other cells. However, only a small number of all possible cell–cell contacts of ILC2 with other lung populations have been studied so far, as depicted in Figure 2, and will be further discussed below.

### ICOS–ICOS-L

Inducible T cell costimulator (CD278) belongs to the CD28 family and is an important molecule in T cell signal transduction (91, 92). ICOS is expressed by mouse and human ILC2 independent of their location, at steady state but also upon stimulation. Deficiency of ICOS or its ligand ICOS-L leads to reduced numbers of ILC2 in the lungs (and small intestine) in combination with lower surface expression of KLRG1 (93). Moreover, ICOS and ICOS-L-deficient mice show reduced levels of pulmonary ILC2 upon intranasal IL-33 stimulation (93, 94). ICOS deficiency in ILC2 in the lungs has also been linked to reduced survival, cytokine production, and pSTAT5, all key for efficient function and signal transduction of ILC2 (94). In the lungs, both, Treg cells and ILC2 are rare at steady state, but can be located at similar sites (8). Interestingly, Tregs have been reported to suppress ILC2 function *via* ICOS and ICOS-L interaction. This interaction appears to take place independent of their location since it was observed in the lungs as well as in visceral adipose tissue (8). However, it is not yet known which signals direct the colocalization of Tregs and ILC2 *in vivo*. Another report investigated the potential of ILC2 suppression by Tregs *via* ICOS–ICOS-L interaction (95). Interestingly, major differences of the suppressive potential of inducible Tregs (iTregs), and natural Tregs (nTregs) were observed. Only iTregs but not nTregs suppressed ILC2 and lead to reduced cytokine secretion by ILC2 (95).

### Programmed Death 1 PD-1-PD-L1/PD-L2

Programmed death 1 (CD279) is another member of the CD28 family like ICOS and has been described as an important negative regulator of T cells (96). Recently, PD-1 treatment has been successfully used in cancer therapy (96). Both ligands for PD-1, PD-L1, and PD-L2, are expressed on various immune and non-immune cells (96). Importantly, ILC2 biology has recently been shown to also be influenced by PD-1 and its ligands. PD-1 is a key factor for ILC2 development and function and is expressed on mature ILC2 as well as on ILC2 progenitors (76, 97). PD-1 negatively regulates KLRG1^+^ ILC2 by inhibiting STAT5 phosphorylation, leading to reduced ILC2 proliferation and cytokine production (97). Deficiency in or blocking of PD-1 results in enhanced ILC2 effector function (97). PD-1 and PD-L1 are upregulated on ILC2 upon immune challenge including IL-33 stimulation (97) or *N. brasiliensis* infection (98). The latter study identified that ILC2 interact *via* PD-L1 with PD-1 on Th2 cells and promote their type 2 effector function (98). Thereby another important regulatory mechanism of ILC2 and their adaptive counterpart, the Th2 cells was identified. Moreover, the importance of the induction of PD-1 or PD-L1 on ILC2 by probably local cells is key to trigger type 2 immune responses.

### KLRG1–E-Cadherin

Killer cell lectin-like receptor subfamily G member 1 is expressed on T cells and NK cells and binds to cadherins (E, N, and R). In contrast to N- and R-cadherins, which are expressed by the nervous system, E-cadherin can be found on epithelial cells as well as on Langerhans cells of the skin (99). A suppressive effect of the interaction of E-cadherin with KLRG1 on human skin ILC2 has been shown, resulting in reduced proliferation and cytokine expression as well as downregulation of GATA3 (100). Pulmonary KLRG1^+^ ILC2 may interact with E-cadherin expressed on lung epithelial cells, especially as part of the adherens junctions, which are located basolateral below the tight junctions and are part of the intercellular junctions formed by the lung epithelium (62, 101). Especially of interest is that high KLRG1 expression has been reported as a surface expression characteristic of iILC2 in the lungs (43). In humans, E-cadherin loss and presence in the sputum correlates with asthma severity (102). However, the exact mechanisms and the downstream signaling pathways within ILC2 upon KLRG1 ligation are not yet understood. Moreover, whether pulmonary ILC2 are able to interact with N- and R-cadherins expressed by the nervous system *via* KLRG1 still remains elusive.

### MHC Class II (MHC-II)–TCR

Different populations of ILC, including ILC2, have been reported to express or upregulate MHC-II upon activation (2, 103, 104). However, the role of MHC-II activation for ILC2 function was just recently deciphered (105, 106). ILC2 express MHC-II upon systemic IL-33 activation to a various extent depending on their location (106): pulmonary ILC2 show less MHC-II expression compared with small intestinal ILC2. ILC2 express functional MHC-II and are able to process and present antigen (106). However, MHC-II expression on ILC2 is only stable *in vivo* and ILC2 loose MHC-II cell surface expression when cultured *ex vivo* for longer periods of time (106). The reasons for that are not yet understood. However, the importance of MHC-II on ILC2 was shown to be independent of the expression levels of MHC-II (106). In addition to systemic administration, intranasal administration of IL-33 induces MHC-II expression on a subpopulation of pulmonary ILC2 (106). Interestingly, despite its heterogeneous expression of MHC-II, the resulting ILC2 population is able to present antigen to T cells. Moreover, IL-2 secreted by T cells further supports type 2 cytokine secretion and proliferation of ILC2 in co-culture experiments (105, 106). Thus, MHC-II on intestinal ILC2 is important to amplify type 2 immune responses in intestinal infections (*N. brasiliensis*). Further research will elucidate whether this mechanism also comes into play for the regulation of beneficial and detrimental type 2 immune responses in the lungs.

## Indirect Interactions of ILC2 with other Cells of the Pulmonary Environment

In addition to direct cell surface receptor–ligand interactions of ILC2 with other non-hematopoietic and/or hematopoietic cells, ILC2 sense their microenvironment and can be regulated by certain cytokines including IL-25 and IL-33 and other immune mediators, which has been recently reviewed (107). However, expression, processing, and release of mediators regulating ILC2 and their cellular origin at steady state and upon immune challenge are poorly investigated and further research is needed to determine their detailed activation mechanism in the pulmonary environment (Figure 2). In addition, cells activating ILC2 are no longer limited to epithelial, endothelial or hematopoietic origin since neurons have been recently added as important regulators of ILC2.

### Regulation of Ilc2 by Neurons

#### Neuromedin U (NMU)

Three recent reports demonstrated that the neuropeptide NMU is a potent activator of ILC2 in the intestinal and respiratory tract (41, 87, 108). NMU is expressed by cholinergic neurons that use the neurotransmitter acetylcholine to transmit impulses. Cholinergic neurons are part of the enteric nervous system, the parasympathetic vagal nerve, and the thoracic dorsal root ganglia (described above). NMU signals *via* the receptors NMUR2, which is mainly expressed on nerve cells, and NMUR1, present on ILC2, their progenitors, and to a much lower degree on T cells (41, 87). NMU can be induced by *N. brasiliensis* excretory/secretory products (NES), alarmins (IL-33), and toll like receptor ligands (LPS) in a-MyD88-dependent manner (LPS) (87). Pulmonary ILC2 express NMUR1 at steady state and upon IL-25 stimulation. By contrast, NMUR1 was shown to be downregulated upon IL-33 stimulation (41). Interestingly, NMU and IL-25 act synergistically to upregulate IL-5 and IL-13 mRNA and protein levels when given intranasally over several days (41). NMU alone was shown to be more potent to induce IL-5 and IL-13 mRNA in intestinal than in pulmonary ILC2 in short-term *ex vivo* stimulations (87). NMU signals *via* ERK1/2 and induces Ca^2+^ influx followed by calcineurin and NFAT activation (87). NMU is also induced in the lung and gut during *N. brasiliensis* infection and NMUR1^+^ ILC2 are superior to NMUR1^−^ ILC2 in fighting off *N. brasiliensis* infection (108). Thus, NMU is an important activator of pulmonary ILC2 (41, 87, 108).

#### Vasoactive Intestinal Peptide (VIP)

The neuropeptide VIP has been initially described as a polypeptide isolated from the small intestine with diverse effects on different systems such as cardiovascular and respiratory systems (109). VIP is expressed by neurons of the central and peripheral nervous system and can be transmitted by VIP receptor type 1 (VPAC1) or VIP receptor type 2 (VPAC2), which are differently regulated dependent on cell type and activity state (110, 111). Interestingly, intestinal and pulmonary ILC2 express VPAC1 and VPAC2 and release IL-5 when they are cultured with IL-7 and VIP- or VPAC2-specific agonist (71). An important link between afferent neurons, VIP, ILC2, and T cells was reported by Talbot and colleagues (112). IL-5 released by ILC2 stimulates nociceptors on afferent neurons and induces the release of VIP, which signals *via* VPAC2 and triggers ILC2 and subsequently T cells to induce more IL-5 and thus creating a type 2 inflammatory feed forward loop highly depending on the neuro-immune axis (112).

### Mediators Released by ILC2

Group 2 innate lymphoid cells also actively shape their microenvironment by the release of several different cytokines mainly IL-5 and IL-13 but also IL-9, IL-10, and amphiregulin (Areg). The secretion of these immune mediators can be beneficial but also detrimental for the host and therefore a well-balanced and fine-tuned release by ILC2 cells is needed.

#### IL-5

Upon activation, ILC2 are able to secrete IL-5, which is important for eosinophil homeostasis (71) and B cell function (1). Both B-1 and B-2 cells are important and prominent populations in the lungs. The production of IgA upon co-culture of mesenteric ILC2 and splenic B cells has been shown to be IL-5 dependent (1). In addition, induction of proliferation as well as IgA, IgM, IgE, and IgG1 secretion by B-1 and B-2 cells by peripheral (peritoneal cavity, spleen) and pulmonary ILC2 was observed in *ex vivo* co-cultures (113). Moreover, pulmonary ILC2 trigger especially IgM secretion by B cells *in vivo* upon NP-Ficoll administration in an IL-5-dependent manner (113). However, how B cells and ILC2 interact within their pulmonary environment *in vivo* still needs further investigation. Moreover, ICOS^+^ ILC2 play a beneficial role through their secretion of IL-5 in a bleomycin model in the lungs (114). Interestingly, especially the timing of IL-5 secretion appears to be crucial for the beneficial effect in this disease model, (114). However, future experiments will be needed to decipher the cellular and molecular cascade triggered by IL-5 to support tissue restoration and homeostasis.

#### IL-9

The expression of the IL-9 receptor by ILC2 has been reported since their detailed description in 2010 (3), and the autocrine role of IL-9 on ILC2 function was deciphered by using IL-9 reporter and subsequently IL-9 fate mapping mice (115, 116). IL-9 reporter mice (*IL9^Cre^R26R^eYFP^*) were used to initially investigate the origin of this cytokine in an IL-33-dependent, papain-induced lung inflammation model where IL-9 production was restricted to innate lymphoid cells (defined as lineage negative, CD45^+^, Thy1^+^ with mixed expression of ST2, CD25, MHCII, and Sca-I). These ILC2-like cells expressed IL-9 only transiently and switched to produce type 2 signature cytokines IL-5 and IL-13. Moreover, IL-33 but not IL-25 induced IL-9 competent ILC. IL-9 production by ILC was induced by IL-2 provided by adaptive immune cells, highlighting again the close relationship between innate and adaptive lymphoid cells. In additional work, IL-9 fate mapping and IL-9R-deficient mice were investigated during *N. brasiliensis* infection and in the latter, less type 2 signature cytokines and Areg were detected indicating that IL-9 is key for efficient ILC2 cytokine production and effector functions. Moreover, IL-9-induced upregulation of BCL3 in these cells is important for their survival (116). Thus, IL-9 acts in an autocrine manner and is important for ILC2 biology. Importantly, IL-2 has been reported to act as a cofactor on ILC2 and increases cell survival and proliferation by triggering NF-κB activation and gene transcription *via* p65 translocation (117). Roediger and colleagues investigated the model of eosinophillic crystalline pneumonia, which spontaneously occurred in Rag-deficient mice in their animal facility. Since several challenges of Rag-deficient mice by IL-2 increased levels of ILC2 (88), CXCR6^+^ ILC2 were investigated and identified as predominantly located in the perivascular in the lungs. Moreover, IL-2 alone did not affect cytokine expression but survival and proliferation and works synergistically with IL-33 to enhance type 2 cytokine expression. Interestingly, next to T cells, Lin^–^ CD90^hi^ CD2^+^ ILC, potentially ILC3, but no myeloid population including dendritic cells and eosinophils were identified as IL-2 producers in the lungs (117). IL-2 and IL-9 are closely interlinked in directing ILC2 biology and enhanced IL-9 expression is linked to an asthma-like phenotype in mice and humans underscoring the importance of these cytokines (118–121).

#### IL-10

The recently described population of regulatory ILC are able to produce IL-10 (122); however, IL-10 production by ILC2 has already been reported (2). Recently, IL-10-producing ILC2 have been observed upon IL-33 or papain challenge in IL-10 reporter mice (123). Interestingly, IL-10 production of ILC2 is transient and decreases after weeks. Although these IL-10-producing ILC2 have a unique profile, they keep ST2 receptor upregulated and do not express T-bet (123).

#### IL-13

With the release of IL-13, ILC2 target non-immune and immune cells in the lungs. Through IL-13 release, ILC2 can initiate mucus secretion but also goblet cell hyperplasia. Whereas controlled mucus secretion is needed to repel particles from the lungs, overproduction of IL-13 leads to goblet cell hyperplasia overexpression of mucus and can thereby also negatively affect tight junctions in the lungs (124). However, the exact pattern of IL-13 receptors on cells of pulmonary origin at steady state and during immune challenge is not yet fully understood. Moreover, IL-13 can induce smooth muscle contraction (125). In addition, ILC2 can prime alveolar macrophages during ontogeny by release of IL-13 into a type 2 immune cell phenotype (126). In addition, ILC2 can target dendritic cells and propagate their migration from the lungs to the LNs in an IL-13-dependent manner (127). Thus, ILC2 are able to influence and trigger innate and adaptive pulmonary type 2 immune responses through release of IL-13.

#### Amphiregulin (Areg)

Group 2 innate lymphoid cells contribute to pulmonary wound healing upon influenza infection *via* the secretion of Areg (14), which belongs to the family of epidermal growth factors (EGF) and signals *via* the EGF receptor (EGFR) (128). Although the detailed expression pattern of EGFR on pulmonary cells is not yet completely elucidated, EGFR has been reported to be expressed by non-hematopoietic and by hematopoietic cells (129). Whereas wound healing and the initiation of mucus secretion is beneficial in some respiratory diseases, it may also be a disadvantage in diseases with enhanced, adverse, and overproduction of mucus (52). Moreover, enhanced EGFR stimulation or signaling has also been implicated to be detrimental in asthma (130). In addition to ILC2, pulmonary Tregs are also able to secrete Areg upon influenza virus infection independent of TCR signaling (131). As such, innate Areg expression by Tregs and ILC2 constitutes an important mechanism to promote wound healing and tissue homeostasis after pulmonary challenge.

## Lung ILC2 Exert Important Effector Functions in Pulmonary Diseases

### Respiratory Virus Infections

Lung infections are the most prevalent infections independent of the economic status of a country and thereby represent a significant disease burden (132). Respiratory virus infections have a high infection and mortality rate worldwide with influenza virus infections alone accounting for approximately 250,000 deaths each year (133). Infections with respiratory viruses can affect the upper [rhinovirus and respiratory syncytial virus (RSV)] as well as the lower respiratory tract [(para-)influenza virus] and often occur in combination with asthma and asthma exacerbations (134). Mice are often used to study respiratory virus infections and the virus is in general administered intranasally to anesthetized mice. Importantly, ILC2 have been shown to be induced upon respiratory virus infections in mice and humans (7, 12, 14, 135–138) and induce an asthma-like phenotype in mice even in the absence of adaptive immunity (7).

#### Influenza A Virus (IAV)

Influenza A virus infection has been shown to induce IL-33 and thereby elicit ILC2 (7, 14, 135). In an infection model using IAV (H3N1 strain), it was shown that ILC2 drive airway hyper-responsiveness (AHR) independently of the adaptive immune response. In this model, alveolar macrophages have been reported to be an important source of IL-33, while eosinophils have not been induced early in infection (7). Another report by Monticelli et al. observed an increase of ILC2 in combination with eosinophilia and identified the contribution of ILC2-derived Areg in wound healing upon infection with a mouse-adapted recombinant H1N1 IAV strain (14). We have recently shown that H1N1-PR8 IAV strain induced eosinophilia as well as neutrophil recruitment and pulmonary type 2 immunopathology. Furthermore, we observed that this immunopathology is significantly increased in interferon receptor 1-deficient animals and identified type I interferon as an important negative regulator to restrain ILC2 upon pulmonary viral infection (135).

#### Rhinovirus

Rhinovirus (RV-16) induces IL-25 mRNA and protein levels in asthmatic patients when compared with healthy individuals (137). The induction of a population of non-T non-NK cells with ST2 and ICOS expression was further reported, which probably corresponds to ILC2 (137). Moreover, rhinovirus (RV-16) infection has been shown to induce IL-33 in asthmatic patients together with a type 2 immune signature *in vivo*. In addition, human Th2 and ILC2 cells can be triggered to secrete type 2 cytokines upon *ex vivo* stimulation with infected bronchial epithelial cells (138). Moreover, rhinovirus (RV1B, propagated in HeLa cells) can induce IL-25, IL-33, and TSLP protein expression and release, thereby triggering ILC2 expansion and activation in young mice (136, 139). Exogenous IFN-γ decreases goblet cell hyperplasia, mucus production, and type 2 signature cytokine expression in the lungs through restraint of pulmonary ILC2 in immature mice (140). Thus, elicitation of ILC2 may depend on a combined induction of IL-25, IL-33, and TSLP in both mouse and humans upon rhinovirus infection.

#### Respiratory Syncytial Virus

Respiratory syncytial virus infections are often linked to asthma exacerbations especially in children. Increased numbers of ILC2 are highly dependent on IL-33 in young mice upon RSV infection (strain A2, propagated in Vero cells) (141). In adult mice as well, ILC2 are elicited upon RSV infection (strain 01/2-20, human isolate, propagated in Hep-2 cells) and the main source of IL-13 early in the infection leading to AHR, goblet cell hyperplasia, and increased mucus production (142). Furthermore, TSLP, probably released by epithelial cells, has been shown to be important for ILC2 activation in this model (142). Interestingly, RSV is able to trigger ILC2 and ILC3 accumulation in the lungs in STAT1-deficient animals resulting in a mixed type 1 and type 2 immune response (143), a phenotype, which is observed sometimes in human asthma (144).

### Helminth Infections

Helminth infections are the most common infections worldwide with approximately 1.5 billion people affected, which equals approximately one-fourth of the world’s population (145). Most helminth infections are not life-threatening for humans, but represent a massive health and economic burden. Type 2 immune responses are essential to efficiently expel the worm and protect from re-infections and ILC2 were shown to participate in and amplify these immune responses.

#### *Nippostrongylus brasiliensis* 

*Nippostrongylus brasiliensis*, a natural rodent helminth, is often used as a model of helminth infections. Upon subcutaneous injection of mice with worm larvae (L3), the larvae migrate into the lungs, molt, get coughed up, swallowed and then reach the intestine (146). ILC2 were initially identified in studies using *N. brasiliensis* as a helminth infection model (1–3). Early on during the infection, ILC2 are induced and responsible for the induction of type 2 signature cytokines, especially IL-13 (1–3). IL-13 release by ILC2 is essential for clearance of the infection since lack of IL-13 results in inefficient worm expulsion and transfer of wild-type ILC2 into IL-13-deficient mice can restore worm clearance (2). In addition, eosinophil counts significantly increase in the lungs upon *N. brasiliensis* infection (2, 3, 147). At the early stage of infection, the lung tissue is extremely fragile, as demonstrated by the increase of red blood cells in the bronchiolar alveolar lavage (147, 148). Using reporter mice, pulmonary ILC2 were identified as the early source for IL-13 and IL-9 during *N. brasiliensis* infection (3, 71, 116). As IL-9 is an important autocrine cytokine that regulates fitness of ILC2, ILC2 and eosinophil levels were found to be reduced in IL-9 receptor-deficient mice (116) (see also paragraph about IL-9). Moreover, the recently identified nILC2 and iILC2 in the lungs are both induced upon *N. brasiliensis* infection albeit with different kinetics: iILC2 are induced early on and nILC2 are dominant in the lungs at late timepoints post infection, suggesting that iILC2 may act as transient progenitors of nILC2 in this setting (43).

### Bacterial Infections

Bacterial infections and their link to ILC2 have not yet been addressed in many settings. Two models of bacterial infections, *Haemophilus influenzae* and *Staphylococcus aureus* have been reported to induce a decrease in GATA3 expression in pulmonary ILC2 similar to what has been reported in influenza infection (85). However, role(s) of pulmonary ILC2 in bacterial infections and associated immunopathologies still need to be further elucidated.

### Allergen-Driven Disease Models

Since the description that ILC2 are able to rapidly secrete large amounts of type 2 signatures cytokines upon activation, their role in pulmonary allergic reactions and asthma models has been studied extensively.

#### House Dust Mite

Allergic reactions to HDM are a very common clinic manifestation in allergic patients (149). HDM can also induce allergic reactions in mice when administered intranasally and is therefore used as a model system to study cellular and molecular mechanism of allergic asthma (AA) (150). HDM extract is a mixture of different components including Der p1 (endopeptidase 1/mite) and Der p2 (TLR4 agonist) as well as serine and cysteine proteases, exo- and endochitinases and endotoxin (LPS) (151). Collectively, these mixtures have been shown to induce both innate and adaptive immune responses including ILC2, Th1, Th2, and Th17 in combination with eosinophils and neutrophils (152, 153). Dose, kinetics and number of administrations as well as the route of administration were shown to influence the outcome of the pulmonary immune response (152, 153). However, ILC2 and T cells follow similar kinetics upon HDM administration and T cells are required for complete ILC2 function in this model (153).

#### Papain Model

Papain is a cysteine protease present in papaya (and other fruit such as pineapple) and has been used in food and drug industries as meat tenderizer but also as tooth whitener in toothpaste and mints. Upon long-term exposure to papain dust, workers in these industries have been reported to develop papain induced asthma in combination with papain specific IgE antibody responses (154). Papain is able to induce an IL-33- and TSLP-dependent type 2 immune response including eosinophilia independent of adaptive immunity in mice (66, 155). Early upon intranasal papain administration, pulmonary ILC2 are the dominant source of IL-5 and IL-13 (66). Dependent on the amount of papain and the number of administrations, increasing levels of NKT and T cells can be detected in the lungs but these seem to be independent of the fitness of pulmonary ILC2 (156). If early (day 0 and 1) and late (day 13 and 20) administration of papain is combined, the support for dendritic cell-dependent priming of Th2 cells by ILC2-derived IL-13 has been reported (127). Thus, papain is an important model to study IL-33-dependent ILC2 function in the pulmonary environment (66).

### Cytokine Administration Models to Study ILC2 Biology

Cytokine administration models (IL-33 or IL-25) are commonly used to study ILC2 biology as they mimic acute type 2 immune responses. These models are less physiological than allergen-dependent or infection models but their advantage is that they use the controlled activity of specific cytokines or cytokine combinations. Interestingly, IL-33 has been reported to be a more potent trigger of type 2 immune responses than IL-25 upon intranasal administration (42). However, intranasal administration of NMU in combination with IL-25 was shown to greatly enhance type 2 immune responses (41).

## Human ILC2 and Asthma

As soon as adverse effects of ILC2 were reported in mouse models of pulmonary disease, the concept emerged of establishing ILC2 prevalence in peripheral blood as a biomarker for diagnostic purposes in lung diseases. Increased levels of IL-33 and ILC2 numbers (Lin^−^IL-7Ra^+^FceRI^−^IL-33R^+^) could be detected in bronchioalveolar lavage of asthma patients when compared with disease controls (patients with a disease history) (157). A different study analyzed ILC2 prevalence (gated as Lin^−^CD127^+^CRTH2^+^ or Lin^−^CD127^+^CD44^high^) in the peripheral blood of allergic asthma (AA) and allergic rhinitis (AR) patients compared to those of healthy donors (158). In general, ILC2 sorted from PBMCs released large amounts of IL-5 and IL-13 when stimulated with IL-2 and IL-33 but only negligible amounts of IL-4. Type 2 cytokine release of PBMCs from AA was increased compared with AR patients upon stimulation. However, PBMCs from healthy donors also secreted a robust level of type 2 signature cytokines but ILC2 prevalence per mL blood was slightly increased in AA patients compared with allergic and healthy donors. Another study compared ILC2 levels (gated on Lin^−^CD45^+^CD127^+^ST2^+^) in blood and sputum of severe asthma, steroid-naïve atopic asthma with healthy controls (159). ILC2 levels as well as type 2 signature positive ILC2 were increased in severe asthma compared with mild asthma patients and eosinophilia was positively correlated. Interestingly, high-dose corticosteroid did not completely reduce ILC2 numbers and signature cytokines, which might be due to TSLP-induced STAT5 phosphorylation in ILC2 which is non-steroid reversible (160). Another report investigated ILC2 (Lin^−^CRTH2^+^CD127^+^) in eosinophilic and non-eosinophilic asthmatic patients, observing a correlation of high ILC2 levels with eosinophilic asthma (161). Asthma is a chronic, heterogeneous disease characterized by airway inflammation and hyper-responsiveness and is more a syndrome than a disease with one specific pattern. Moreover, asthmatic patients have been reported with higher neuropeptide levels, adding once more the neuro-immune axis (162). Thus, ILC2 characterization in different asthmatic subgroups and their comparison is needed and the use of ILC2 levels as a biomarker needs to be carefully evaluated for each subgroup.

## Counteracting ILC2 to Ameliorate Pulmonary Diseases

Group 2 innate lymphoid cells are critical initiators and amplifiers of type 2 immune responses. Therefore, understanding the regulation of ILC2 will help to understand how type 2 immune responses can be regulated in general. Type I and II interferons have been recently shown to be important in restricting ILC2 effector functions (8, 12, 135). Importantly, also IL-27, a member of the IL-12 cytokine family, is able to suppress ILC2 function (12, 135, 163). These reports showed that both interferons and IL-27 restrain ILC2 by STAT1 activation and thereby act as negative regulators of ILC2-mediated responses (12, 135). Interestingly, IL-27 can also restrict ILC2 *via* activation of STAT3 (163). However, STAT1 activation has been recently reported to not only restrain ILC2 but also exhibit negative regulatory potential on ILC3 (143). Interestingly, the IL-10 receptor is expressed on ILC2 from the mesentery, but no inhibitory effect of IL-10 was observed *ex vivo* (1, 12). However, pulmonary ILC2 upon challenge with papain or IL-33 are restrained when cultured *ex vivo* with IL-10 (164). This is of special interest as the recently described regulatory ILC execute their suppressive anti-inflammatory functions independent of Foxp3 but *via* the release of IL-10 (122). As such, regulatory ILC might constitute important negative regulators of ILC2. Importantly, negative regulation of ILC2 by IL-10 was shown to be dependent on their stimulation by IL-33 (164), further highlighting the importance of the cytokine milieu for ILC2 function. ILC2 sense and express the respective receptors for IL-33, namely ST2 and IL-1 receptor accessory protein and TSLP, TSLP receptor chain together with the IL-7Rα chain (107). IL-33 signal transduction results in MyD88-dependent activation of NF-κB and MAPK pathways, whereas TSLP mainly signals *via* STAT5. In human ILC2, TSLP induces cytokine expression by triggering GATA3 expression through STAT5 phosphorylation (17). Polymorphisms in genetic loci of IL-33, its receptor *ST2* (*IL1RL1*), and *TSLP* have been identified among other genes in genome-wide association studies in asthmatic patients (165) and monoclonal antibodies specific for TSLP have been positively evaluated in asthmatic patients (166, 167). Thus, this might be a therapeutical strategy to restrain ILC2 by capturing ILC2 eliciting effectors such as TSLP; however, plasticity of ILC2 might hinder this approach.

## Summary and Outlook

Since their detailed description in 2010, ILC2 have been studied and followed with huge interest and excitement. In this review, we focused on ILC2 biology and function within their pulmonary environment, giving an overview about lung physiology as well as different disease and allergy models in the lungs in which ILC2 are known to play a role. The pulmonary environment is especially fascinating to study ILC2 biology as they are the predominant ILC population within the lung. Thus, ILC2 play an important role in the initiation but also orchestration of the pulmonary immune response. However, we are just beginning to understand their exact cellular and molecular interactions, their dynamics, and especially their ability of surveillance within the pulmonary tissue at steady state and upon challenge. ILC2 have been mainly reported to play a role in deregulated type 2 immune responses. However, the positive impact of type 2 immune responses has also been proposed (168) and the contribution and the role of ILC2 in this regard has only been started to be elucidated.

## Author Contributions

All authors listed have made a substantial, direct, and intellectual contribution to the work and approved it for publication.

## Conflict of Interest Statement

The authors declare that the research was conducted in the absence of any commercial or financial relationships that could be construed as a potential conflict of interest.
